# A novel 5′-hydroxyl dinucleotide hydrolase activity for the DXO/Rai1 family of enzymes

**DOI:** 10.1093/nar/gkz1107

**Published:** 2019-11-28

**Authors:** Selom K Doamekpor, Agnieszka Gozdek, Aleksandra Kwasnik, Joanna Kufel, Liang Tong

**Affiliations:** 1 Department of Biological Sciences, Columbia University, New York, NY 10027, USA; 2 Institute of Genetics and Biotechnology, Faculty of Biology, University of Warsaw, 02-106 Warsaw, Poland

## Abstract

Modifications at the 5′-end of RNAs play a pivotal role in determining their fate. In eukaryotes, the DXO/Rai1 family of enzymes removes numerous 5′-end RNA modifications, thereby regulating RNA turnover. Mouse DXO catalyzes the elimination of incomplete 5′-end caps (including pyrophosphate) and the non-canonical NAD^+^ cap on mRNAs, and possesses distributive 5′-3′ exoribonuclease activity toward 5′-monophosphate (5′-PO_4_) RNA. Here, we demonstrate that DXO also catalyzes the hydrolysis of RNAs bearing a 5′-hydroxyl group (5′-OH RNA). The crystal structure of DXO in complex with a 5′-OH RNA substrate mimic at 2.0 Å resolution provides elegant insight into the molecular mechanism of this activity. More importantly, the structure predicts that DXO first removes a dinucleotide from 5′-OH RNA. Our nuclease assays confirm this prediction and demonstrate that this 5′-hydroxyl dinucleotide hydrolase (HDH) activity for DXO is higher than the subsequent 5′-3′ exoribonuclease activity for selected substrates. Fission yeast Rai1 also has HDH activity although it does not have 5′-3′ exonuclease activity, and the Rat1-Rai1 complex can completely degrade 5′-OH RNA. An *Arabidopsis* DXO1 variant is active toward 5′-OH RNA but prefers 5′-PO_4_ RNA. Collectively, these studies demonstrate the diverse activities of DXO/Rai1 and expands the collection of RNA substrates that can undergo 5′-3′ mediated decay.

## INTRODUCTION

The chemical composition at the 5′ end of RNAs plays a critical role in all facets of RNA biology, including biosynthesis, processing, transport, and decay (1–5). Enzymes that modify or remove these 5′ ends therefore represent key regulatory inputs into these pathways (6–8). In eukaryotes, the most common modification that occurs on mRNAs is conversion of the nascent 5′ triphosphate end to a 5′ *N*^7^-methylguanosine (m^7^G) cap (1). The m^7^G cap is essential for stability, efficient splicing, nuclear export, and translation. In the cytoplasm, the Nudix hydrolase family of decapping enzymes such as Dcp2 and Nudt16 can initiate 5′-3′ mediated decay by hydrolyzing m^7^G caps and releasing m^7^GDP (9). As a result, these decapping enzymes produce 5′ monophosphate (5′-PO_4_) RNAs for rapid decay by the cytoplasmic processive 5′-3′ exoribonuclease Xrn1 (10,11).

Our earlier studies show that m^7^G capping does not always proceed to completion and a 5′-end capping quality surveillance mechanism exists in the nucleus to remove the incompletely-capped RNAs. This surveillance pathway is mediated by the DXO/Rai1 family of decapping enzymes (12–15), which bears no sequence or structural similarity to the Nudix enzymes. Activity toward incomplete m^7^G caps was first demonstrated with *Schizosaccharomyces pombe* Rai1 (SpRai1), which possesses RNA 5′ pyrophosphohydrolase (PPH) activity (hydrolyzing 5′ triphosphate RNA (pppRNA) to generate pyrophosphate and 5′-PO_4_ RNA) (12) and non-classical decapping activity (releasing GpppN from unmethylated caps) (13). Rai1 forms a stable complex with Rat1 (the nuclear homolog of Xrn1) in yeast (16,17), which also has processive 5′-3′ exoribonuclease activity, thereby coupling decapping with decay. Since then, this decapping activity toward unmethylated caps has been extended to other DXO/Rai1 homologs that have been investigated (18), including the fungal cytoplasmic Dxo1 (14) and mammalian DXO (15).

However, members of the DXO/Rai1 family display distinct activities toward other 5′-end modified RNAs. While mouse DXO has PPH activity, budding yeast Dxo1 cannot hydrolyze pppRNA, and some fungal Rai1 enzymes perform 5′-triphosphonucleotide hydrolase (TPH) activity instead of PPH activity (18). Additionally, Dxo1 and DXO (and some fungal Rai1 enzymes) possess 5′-3′ exoribonuclease activity toward 5′-PO_4_ RNA and can completely degrade RNA independent of Rat1/Xrn1 exoribonucleases (14,15,18). Cap surveillance and exonuclease activities can also be reduced by a point modification within the catalytic site, as is the case in *Candida albicans* Rai1 (18) and *Arabidopsis thaliana* DXO1 (19).

Structural studies showed that DXO/Rai1 enzymes share a common fold and utilize the same catalytic machinery to perform their various activities (12,14,15,18,20). Six conserved sequence motifs (I–VI) (18) form the active site which is located within a deep pocket, and several residues in these motifs bind divalent cations for catalysis. Variable residues within this cavity appear to define their different catalytic activities although it is still not clear in many cases how this takes place (18).

Recently, the catalog of DXO cellular substrates has expanded to include non-canonical nicotinamide adenine dinucleotide (NAD^+^) capped RNAs (20). First discovered in bacteria (21–23), it was later established that RNAs in yeast and humans can also be modified at their 5′ end by NAD^+^ (20,24,25). In contrast to prokaryotic NAD^+^ and eukaryotic m^7^G caps that stabilize RNA, eukaryotic NAD^+^ caps promote decay through DXO mediated removal of the entire NAD^+^ moiety (deNADding) (20). The crystal structures of DXO and Rai1 in complex with the NAD^+^-capped RNA mimic, 3′-phospho NAD^+^ (3′-NADP^+^), demonstrated that the same active site is used to perform the deNADding reaction and that this active site can accommodate the entire NAD^+^ cap (20).

The recent identification of additional DXO targets engenders the notion that DXO may regulate RNAs with other, less thoroughly studied 5′ ends. While the 5′-PO_4_ group of the substrate is specifically recognized in the active site of DXO for its exonuclease activity (15), here we demonstrate that DXO surprisingly can also catalyze the hydrolysis of 5′-hydroxyl (5′-OH) RNA. In fact, we show that DXO displays higher activity towards 5′-OH RNA than 5′-PO_4_ RNA. The crystal structure of DXO with a 5′-OH RNA substrate mimic at 2.0 Å resolution illuminates the molecular basis for this activity. More importantly, the structure predicts that DXO initially removes a dinucleotide from 5′-OH RNA, and we have confirmed this 5′-hydroxyl dinucleotide hydrolase (HDH) activity by biochemical studies. Finally, we demonstrate that both SpRai1 and *Arabidopsis* DXO1(ΔN194) have HDH activity, and that the yeast Rat1–Rai1 complex is capable of robust 5′-OH exoribonuclease activity due to removal of the 5′-OH end by Rai1 followed by processive decay by Rat1.

## MATERIALS AND METHODS

### Protein expression and purification

Full-length mouse DXO or SpRai1 in pET28a was used to transform *Escherichia coli* BL21 (DE3) Rosetta cells. His-tagged DXO and SpRai1 proteins were produced and purified as previously reported (12). Briefly, DXO/SpRai1 production was induced with 0.3 mM IPTG at 18°C for 18 h. After cell disruption by sonication and centrifugation, the lysate (in a buffer containing 400 mM NaCl, 20 mM Tris (pH 7.5), 20 mM imidazole, 5 mM β-mercaptoethanol (BME) and 1 mM phenylmethanesulfonylfluoride (PMSF)) was incubated with 2 ml of Ni-NTA superflow resin (Qiagen) with nutation for 1 h. DXO was eluted with 5 ml of buffer containing 250 mM NaCl, 20 mM Tris (pH 7.5), 250 mM imidazole and 5 mM BME and further purified with gel filtration (Sephacryl S-300, GE Healthcare) chromatography (in buffer containing 250 mM NaCl, 20 mM Tris (pH 7.5) and 2 mM DTT). DXO/SpRai1 proteins were supplemented with 5% (v/v) glycerol and concentrated to 10 mg/ml before being frozen in liquid nitrogen.

To produce Rat1 and the Rat1-Rai1 complex, full-length SpRat1 in pET24d (producing Rat1 protein with a C-terminal hexa-histidine tag) and Rai1 in pET26b (producing Rai1 with no affinity tag) were separately used to transform *E. coli* BL21 (DE3) Rosetta cells. After induction with 0.3 mM IPTG and growth at 18°C for 18 h, cells producing Rat1 were lysed and sonicated with or without cells producing Rai1 to generate Rat1 protein alone or Rat1–Rai1 complex respectively (12). Rat1/Rat1–Rai1 proteins were then purified using the same protocol as that for DXO/SpRai1.

### Crystallization, data collection and structure determination

DXO crystals were obtained using the hanging-drop vapor diffusion method at 20**°**C with a reservoir solution containing 21–26% (w/v) PEG 3350 (12). To generate 5′-OH U(S)6 RNA, pU(S)6 RNA was incubated with 5 units of CIP at 37°C for 2 h. Crystals were soaked with 20 mM CaCl_2_ for 1 h followed by soaking in 10 mM 5′-OH U(S)6 (in a fresh drop) for an additional hour at 20**°**C in buffer containing 25% (w/v) PEG 3350. Soaked DXO crystals were cryo-protected with 25% (w/v) PEG 3350 and 25% (v/v) ethylene glycol before being flash frozen in liquid nitrogen for diffraction analysis and data collection at 100 K.

X-ray diffraction data were collected at the Advanced Photon Source (APS) beamline 24-ID-E. The wavelength used was 0.97918 Å. The crystal to detector distance was 250 mm, and each image covered 0.25° rotation of the crystal. The diffraction images were recorded on a Dectris Eiger 16M detector, processed and scaled with standard parameters using the XDS program (26). Reflections from 800 images were included in the final data set. The crystal was isomorphous to that reported earlier (12) and belongs to space group *P*2_1_ with 1 molecule in the asymmetric unit. The structure refinement was performed using PHENIX (27) and manual rebuilding of the atomic model was carried out with the Coot program (28). Rebuilding of the protein model was completed before the RNA was built into the difference electron density map and included in the refinement. The density for the metal ion was assigned as calcium. It was observed only when the crystal was soaked with this metal ion. The crystallographic information is summarized in Table 1.

**Table 1.** Summary of crystallographic information

Structure	DXO in complex with 5′-OH U(S)6 RNA and Ca^2+^
**Data Collection**	
Space group	*P*2_1_
Cell dimensions	
*a*, *b*, *c* (Å)	50.2, 87.8, 53.9
α, β, γ (°)	90, 112.3, 90
Resolution (Å)	49.8–2.0 (2.07–2.0)
*R*_merge_ (%)	3.5 (44.7)
I/σI	12.3 (2.5)
Completeness (%)	99.1 (96.7)
No. of reflections	56459
Redundancy	1.9 (1.9)
**Refinement**	
Resolution (Å)	49.8–2.0 (2.07–2.0)
*R*_work_/*R*_free_ (%)	18.9 (33.8) / 22.2 (40.4)
Number of atoms	3168
Protein	2937
Ligand/Ion	103
Water	128
*B*-factors (Å^2^)	41.5
Protein	40.7
Ligand/Ion	60.3
Water	45.0
r.m.s.d.	
Bond lengths (Å)	0.005
Bond angles (°)	0.74
Ramachandran plot statistics	
Favored	97.5
Allowed	2.5
Outliers	0.0

The average B value for the first two nucleotides of the RNA (51 Å^2^) is higher than that for the protein (41 Å^2^, Table 1). A refinement with the occupancy of this RNA set at 0.8 produced an average B of 43 Å^2^ for these nucleotides. Weak electron density for a nucleotide at the crystal packing interface was observed, and was modeled as 3′-PO_4_ UMP.

### Assays with fluorescently labeled RNA

A 3′-end FAM-labeled 30-mer G-less RNA (29), P-ACUCACUCACUCACCAAAAAAAAAAAAACC-FAM, and a 34-mer P-GGGAGACCGGCCUCGAGAUCGAUGAUAUCGAAUU-FAM RNA, with a 5′-monophosphate were treated with CIP for 2 h at 37°C to generate 5′-hydroxyl RNA, confirmed by Xrn1 and Xrn2 exoribonuclease assays. 3′-end FAM-labeled 25-mer RNA oligonucleotides, P-ACUCACUCACUCACCAAAAAAAACC-FAM and OH-ACUCACUCACUCACCAAAAAAAACC-FAM were obtained from Future Synthesis.

Exoribonuclease assays were performed at 37°C for the indicated time periods with reaction mixtures containing 30 mM Tris (pH 8.0) (or pH 6.0–9.0 with appropriate buffers as indicated), 50 mM NH_4_Cl, 2 mM MgCl_2_, 1 mM MnCl_2_, 2 mM DTT, 25 μg ml^−1^ BSA, 100 nM 3′-end FAM-labeled RNA and the indicated amount of recombinant DXO/Rai1 proteins. The products were fractionated by a 7M urea, 16% (w/v) polyacrylamide electrophoresis (PAGE) gel in 1× Tris/Borate/EDTA (TBE) buffer and visualized on a Typhoon FLA 7000 (GE Healthcare).

### Assays with radiolabeled RNA

*5′-end hydrolysis assays*. RNA substrate (60 nt: P-AGTCCTCTCTCTCTCCTCTCTCTCCTCTTCCTTCTCTTCCTTCTCCTCTCCTCTTCCTTC) was designed with a T7 ϕ2.5 promoter and a single G nucleotide at the second position to enable the discrimination between mononucleotide and dinucleotide released from the RNA 5′ ends. Radioactively labeled RNA was obtained by *in vitro* transcription with T7 RNA polymerase in the presence of [α-^32^P]GTP (Hartmann Analytics) and AMP (Sigma) to generate pRNA that was further treated with alkaline phosphatase FastAP (Thermo Fisher) for 2 h at 37°C to produce OH-RNA. Reactions were carried out in buffer containing 10 mM Tris (pH 7.5), 50 mM KOAc, 2 mM Mg(OAc)_2_, 2 mM MnCl_2_, 1 mM DTT, 0.1 mM spermine, 20 U RiboLock RNase inhibitor, 20 cps of radiolabeled RNA and indicated amount of recombinant protein. Reactions were stopped with 0.05 M EDTA and resolved on polyethyleneimine-cellulose TLC plates in 0.7 M KH_2_PO_4_ (pH 4.5). Plates were analyzed with PhosphorImager Typhoon FLA 9000.

## RESULTS

### Mouse DXO displays 5′-3′ exoribonuclease activity on 5′-OH RNA

**Figure 1.**
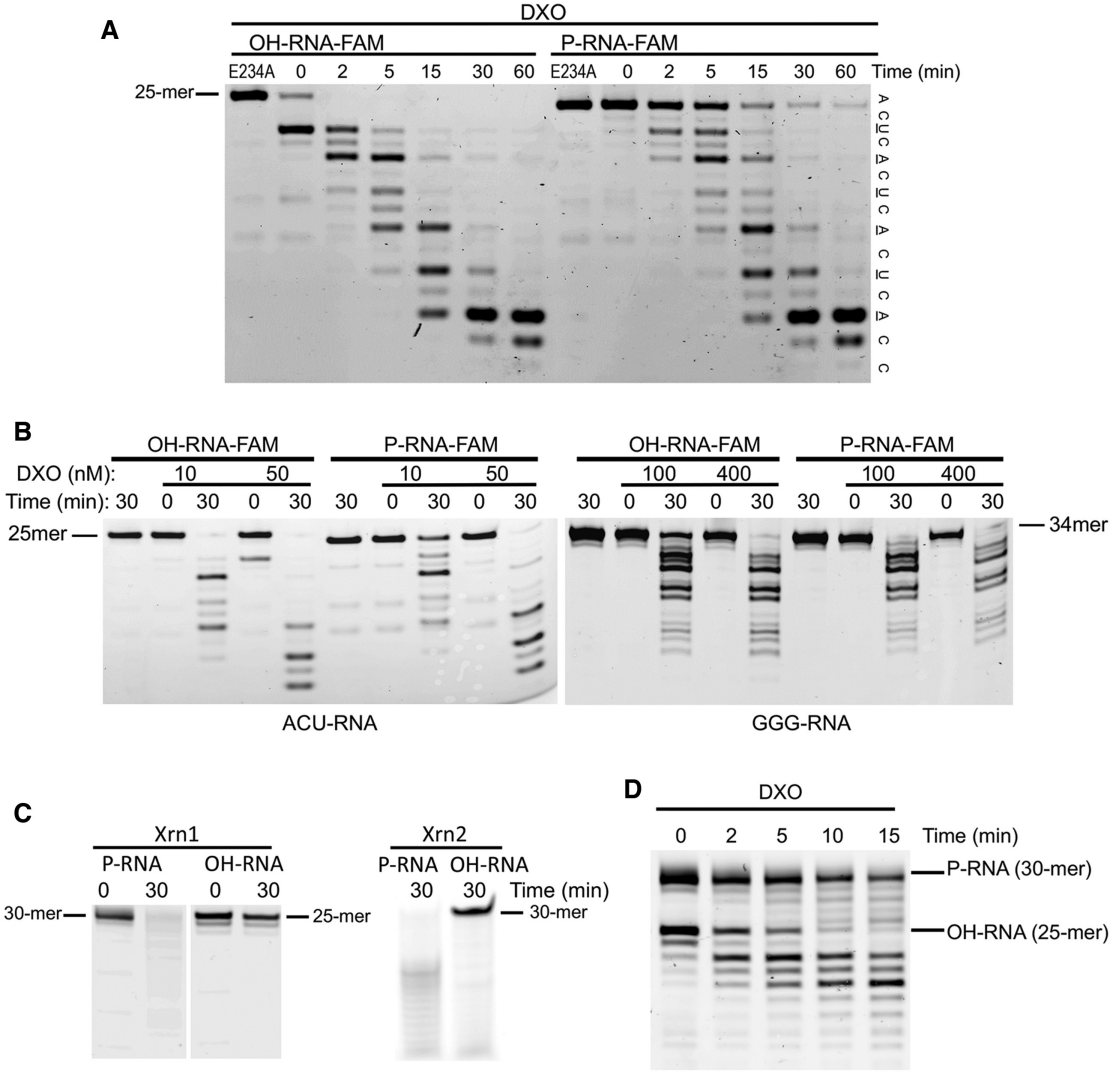
Mouse DXO displays activity toward 5′-OH RNA. (**A**). Mouse DXO (100 nM) shows activity toward 5′-PO_4_ and 5′-OH G-less FAM-labeled RNA substrates. The 5′-OH substrate (OH-RNA) is degraded faster than the 5′-PO_4_ substrate (P-RNA). The 5′ nucleotide of each RNA product is shown on the right, and those showing transient accumulation are underlined. (**B**). Mouse DXO prefers 5′-OH RNA over 5′-PO_4_ RNA G-less substrates, while the opposite is true for substrates with three Gs at the 5′ end. The 5′-PO_4_ (P-RNA) GGG-RNA is digested more efficiently than the 5′-OH (OH-RNA) GGG-RNA. (**C**). 5′-3′ exoribonuclease activity of 0.003 U Xrn1 or 100 nM Xrn2 on the same 5′-PO_4_ or 5′-OH RNA substrates, demonstrating the absence of activity towards 5′-OH RNA. (**D**). Simultaneous incubation of 400 nM DXO with a 30-mer 5′-PO_4_ RNA and a 25-mer 5′-OH RNA.

DXO has distributive 5′-3′ exoribonuclease activity on 5′-PO_4_ RNAs, and this 5′-PO_4_ group is recognized by interactions with Arg132 (conserved motif I) and Gln280 (conserved motif V) (15). Surprisingly, when we tested the activity of DXO toward 5′-OH RNA substrates, we found that the enzyme is active toward them as well. To compare DXO activity toward 5′-PO_4_ RNA *versus* a 5′-OH RNA, we performed decay assays *in vitro* using a 30 nucleotide G-less RNA with a 3′-FAM (6-carboxyfluorescein) label (29). As expected, DXO degraded 5′-PO_4_ RNA, generating multiple intermediates, indicative of its distributive activity (Figure 1A). DXO also hydrolyzed 5′-OH RNA, and appeared to be faster at removing this 5′-end modification than the subsequent 5′-3′ exonuclease activity toward the 5′-PO_4_ RNA (Figure 1A). DXO’s preference for 5′-OH substrate and the rate of its hydrolysis were not affected by pH (pH range 6–9), in contrast to the 5′-3′ exonuclease activity, which was more pronounced at higher pH (Supplementary Figure S1). For both substrates, DXO appeared to be a little slower at removing a 5′ U or A nucleotide than C in the intermediate products, resulting in transient accumulation of intermediates with 5′ U or A. In the case of 5′-PO_4_ and 5′-OH RNA substrates with three consecutive Gs at the 5′ end, the activity toward both was much weaker than that toward the G-less substrate (Figure 1A). More importantly, the preference was reversed with a stronger bias toward 5′-PO_4_ RNA than 5′-OH RNA for GGG–RNA (Figure 1B). As a control, Xrn1 and Xrn2 were only active toward 5′-PO_4_ RNA (Figure 1C), consistent with its dependence on the 5′-PO_4_ modification.

We confirmed the enhanced activity toward 5′-OH G-less RNA by simultaneously incubating DXO with RNAs containing either a 5′-PO_4_ or a 5′-OH group. In order to more clearly distinguish the products, we used a shorter 5′-OH RNA (25 nucleotides) than 5′-PO_4_ RNA (30 nucleotides). The level of the intact 5′-OH RNA decreased faster than intact 5′-PO_4_ RNA, demonstrating that, depending on the composition, RNA with a 5′-OH can be the preferred substrate for DXO at similar RNA concentrations (Figure 1D).

### Structural basis for DXO activity toward 5′-OH RNA

We previously illuminated the mechanism of DXO 5′-3′ exoribonuclease activity toward 5′-PO_4_ RNA releasing single nucleotides (15). The crystal structures of DXO in complex with the 5′-PO_4_ RNA product pU5, and non-cleavable substrate mimic pU(S)6, (containing two phosphorothioate groups, between the first and second and second and third nucleotides) revealed that the RNA body is accommodated in a positively charged pocket. The active site is located near the bottom of this pocket, where divalent cations bind to position the RNA scissile phosphate (between the first and second nucleotide) for nucleophilic attack.

To understand the molecular basis for the catalytic activity of DXO toward 5′-OH RNA, we determined the crystal structure of wild-type mouse DXO in complex with a 5′-OH hexa-nucleotide non-hydrolyzable RNA substrate mimic, OH-U(S)6. OH-U(S)6 was generated by incubating pU(S)6 with calf intestinal alkaline phosphatase (CIP) to remove the 5′-PO_4_ group. As we demonstrated with another RNA, 5′-PO_4_ RNA treated in this way was successfully degraded by DXO but not by Xrn2 (Figure 1C), confirming the removal of the 5′-PO_4_ group. DXO crystals were soaked with OH-U(S)6 RNA and with calcium as the divalent cation to further block hydrolysis. The structure was determined at 2.0 Å resolution and has excellent agreement with the X-ray diffraction data and the expected bond lengths, bond angles and other geometric parameters (Table 1).

**Figure 2.**
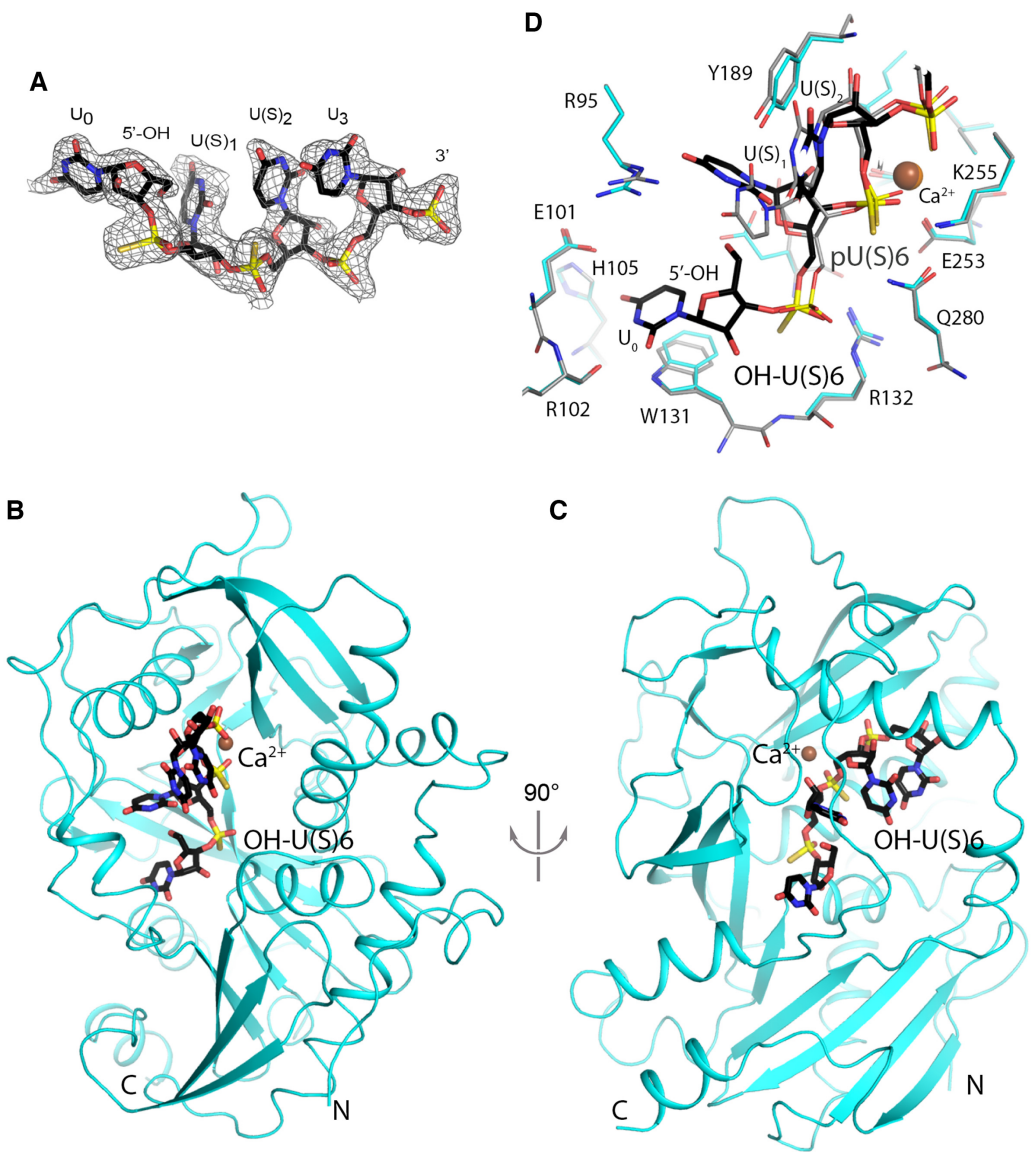
Crystal structure of mouse DXO in complex with 5′-OH-U(S)6. (**A**) Simulated annealing omit *F*_o_ – *F*_c_ electron density at 2.0 Å resolution for 5′-OH U(S)6 RNA, contoured at 2σ. (**B**) Overall structure of mouse DXO (cyan) in complex with 5′-OH-U(S)6 RNA (in black for carbon atoms). (**C**) Overall structure of mouse DXO (cyan) in complex with 5′-OH-U(S)6 RNA, viewed after 90° rotation around the vertical axis. (**D**) Overlay of the active site region of mouse DXO in complex with 5′-OH U(S)6 RNA (in color) with that in complex with pU(S)6 RNA (gray; PDB: 4J7M) (15). The calcium ion from the pU(S)6 structure is shown in orange.

We observed good electron density for four nucleotides of the OH-U(S)6 RNA (Figure 2A) and a calcium ion in the complex with DXO (Figures 2B, C). An additional nucleotide at the 3′ end showed weak electron density and was not modeled. The 5′-end nucleotide contains a hydroxyl moiety, again confirming the removal of the 5′-PO_4_ group during the CIP treatment (Figure 2A). We were able to unambiguously assign the nucleotide positions due to the stronger electron density of sulfur atoms in the phosphorothioate groups compared to oxygen (Figure 2A), which also confirmed that there was no hydrolysis of this RNA during crystallization. The average *B* value for the first two nucleotides of the RNA is 51 Å^2^, higher than that for the protein (41 Å^2^, Table 1), suggesting that the RNA may be at a somewhat lower occupancy (∼80%) in the crystal. Nonetheless, the observed electron density for the RNA (Figure 2A) provides clear indication of its binding mode.

There is electron density for a calcium ion bound to the active site residues (Asp236, Glu253 and Glu192) (Figure 2D), and this density is not observed in crystals not soaked with calcium. However, the calcium ion contacts the phosphorothioate group between the second and third nucleotides of this RNA instead of between the first and second as was observed with pU(S)6 (15) (Figure 2D). This suggests that the scissile phosphate of this 5′-OH RNA is located between its second and third nucleotides, and more importantly, it predicts that DXO generates a 5′-OH dinucleotide as the first product of the hydrolysis. To be consistent with the earlier nomenclature in terms of the nucleotides in the RNA substrate (15), we named the four observed nucleotides in the OH-U(S)6 RNA U_0_, U(S)_1_, U(S)_2_, and U_3_, so that the scissile phosphate is located between U_1_ and U_2_ (Figures 2A, D).

The U_0_ base has π stacking interactions with the mostly conserved Trp131 side chain, and it also makes three hydrogen-bonding contacts: to the backbone carbonyl of Arg102 and the side chain of His105 directly and to the backbone amide of His105 through a water molecule (Figure 2D). The 5′-OH moiety itself is intimately packed against the base of U(S)_1_ (Figure 2D). The presence of a phosphate here would make it impossible to adopt this conformation, which may account for why 5′-PO_4_ RNA cannot bind in this manner.

The overall structure of DXO in the OH-U(S)6 complex is essentially the same as that of the pU(S)6 complex, with rms distance of 0.3 Å for 358 equivalent Cα atoms between the two structures. The conformation of most of the side chains in the active site region is similar between the two structures as well (Figure 2D). However, there are substantial differences in the binding modes of the U(S)_1_ nucleotide between OH-U(S)6 and pU(S)6 (Figure 2D). The position of its 5′ phosphate group of OH-U(S)6 differ by 1.5 Å compared to that of pU(S)6, although the two phosphates can maintain similar interactions with DXO. The U(S)_1_ base moves by ∼3 Å, and the position of the U(S)_1_ base in the pU(S)6 structure would clash with the 5′-OH of the OH-U(S)6 RNA (Figure 2D). In addition, the U(S)_1_ base is in the *syn* configuration in OH-U(S)6 RNA, while it is *anti* in pU(S)6. In comparison, the conformation of the U_2_ and U_3_ nucleotides in OH-U(S)6 RNA is nearly the same as that in pU(S)6.

**Figure 3.**
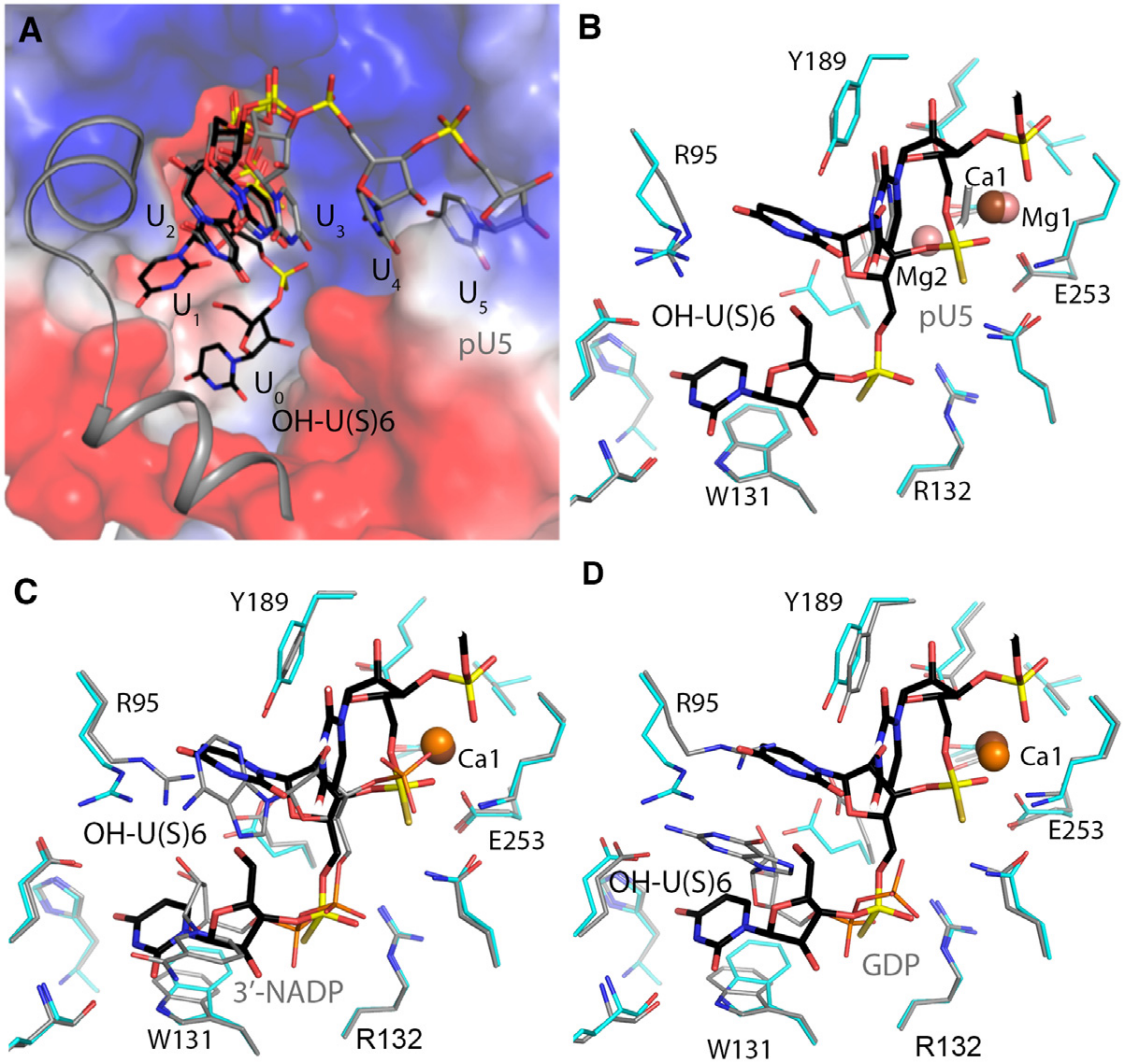
Comparison of 5′-OH U(S)6 RNA binding mode to other DXO ligands. (**A**) The active site pocket. Surface representation of mouse DXO in the active site region, colored by the electrostatic potential (with blue and red indicating positive and negative potential, respectively). Two helices near the opening of the pocket are shown as cartoons for clearer visualization of the pocket. (**B**) Overlay of the active site region of mouse DXO in complex with 5′-OH U(S)6 RNA (in color) with that in complex with pU5 RNA (gray; PDB: 4J7L) (15). The 2 magnesium ions in the pU5 complex are shown in pink. (**C**) Same as in B but with the 3′-NADP complex (PDB: 5ULI) (20). (**D**) Same as in B but with the GDP complex (PDB: 3FQJ) (12).

The U_0_ nucleotide is accommodated deeper within the DXO active site (Figure 3A) and is in the same general region as the nicotinamide diphosphate moiety of the NAD^+^ cap and GDP (Figures. 3B–D) (12,15,20). Most of the DXO side chains in these structures are in the same conformation except for Arg95. Arg95 interacts with the ribose hydroxyls and makes van der Waals contacts with the adenine base in the 3′-NADP complex (Figure 3C) and with the guanine base in the GDP complex (Figure 3D), but it swings out of the way with 5′-OH RNA where it only hydrogen bonds with the U_1_ base. Together these structures demonstrate that DXO can carry out multiple catalytic activities by being able to accommodate and make specific contacts to various orientations of the 5′ modification.

### DXO releases a 5′-dinucleotide from 5′-OH RNA

**Figure 4.**
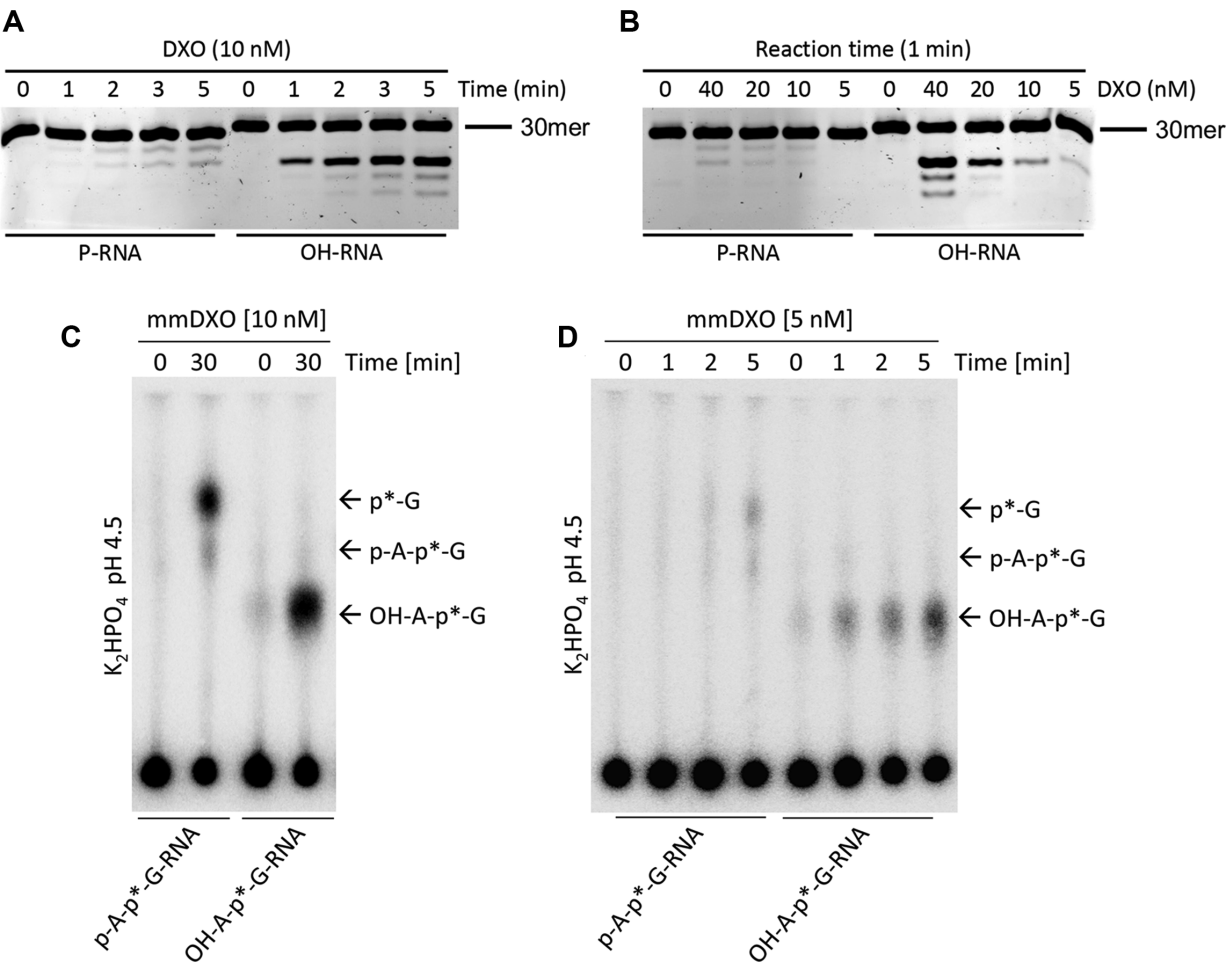
DXO releases a 5′-dinucleotide from 5′-OH RNA. (**A**) Time course monitoring early points of the 5′-3′ exoribonuclease reaction with 10 nM DXO and G-less 5′-PO_4_ RNA (P-RNA) and OH-RNA. (**B**) Product formation after incubation for 1 min of G-less P-RNA and OH-RNA with increasing concentrations of DXO. (**C**) TLC of the exoribonuclease reaction after 30 min using 10 nM mouse DXO with RNAs radiolabeled on the phosphate between the first and second nucleotides. The migration of the 5′ nucleotides are indicated. (**D**) Same as C but a time course using 5 nM mouse DXO.

We next set out to confirm our structural prediction that DXO releases a dinucleotide from 5′-OH RNA as the first product. We performed exoribonuclease assays and monitored the initial cleavage events. 3′-end 6-FAM labeled RNA with either a 5′-PO_4_ or 5′-OH was incubated with DXO, and the products were separated on a denaturing gel that could clearly resolve single nucleotide changes to the RNA. The first RNA product released from 5′-OH RNA migrated at the same position as the second product released from 5′-PO_4_ RNA, suggesting that DXO released a dinucleotide from 5′-OH RNA in the first step of the reaction (Figure 4A). We propose the name 5′-hydroxyl dinucleotide hydrolase (HDH) for this activity. After the initial dinucleotide cleavage from 5′-OH RNA, single nucleotides were released thereafter, signifying that the remaining RNA has a 5′-PO_4_.

To further confirm that DXO releases a 5′-OH dinucleotide leaving behind a 5′-PO_4_ RNA, we used an RNA radiolabeled on the phosphate between the first and second nucleotide, and carried out TLC (thin layer chromatography) to separate the reaction products. Consistent with our crystal structure and gel based assays, DXO released a 5′-OH dinucleotide from 5′-OH RNA but single nucleotides from 5′-PO_4_ RNA (Figures 4B and C).

### DXO homologs also have HDH activity

**Figure 5.**
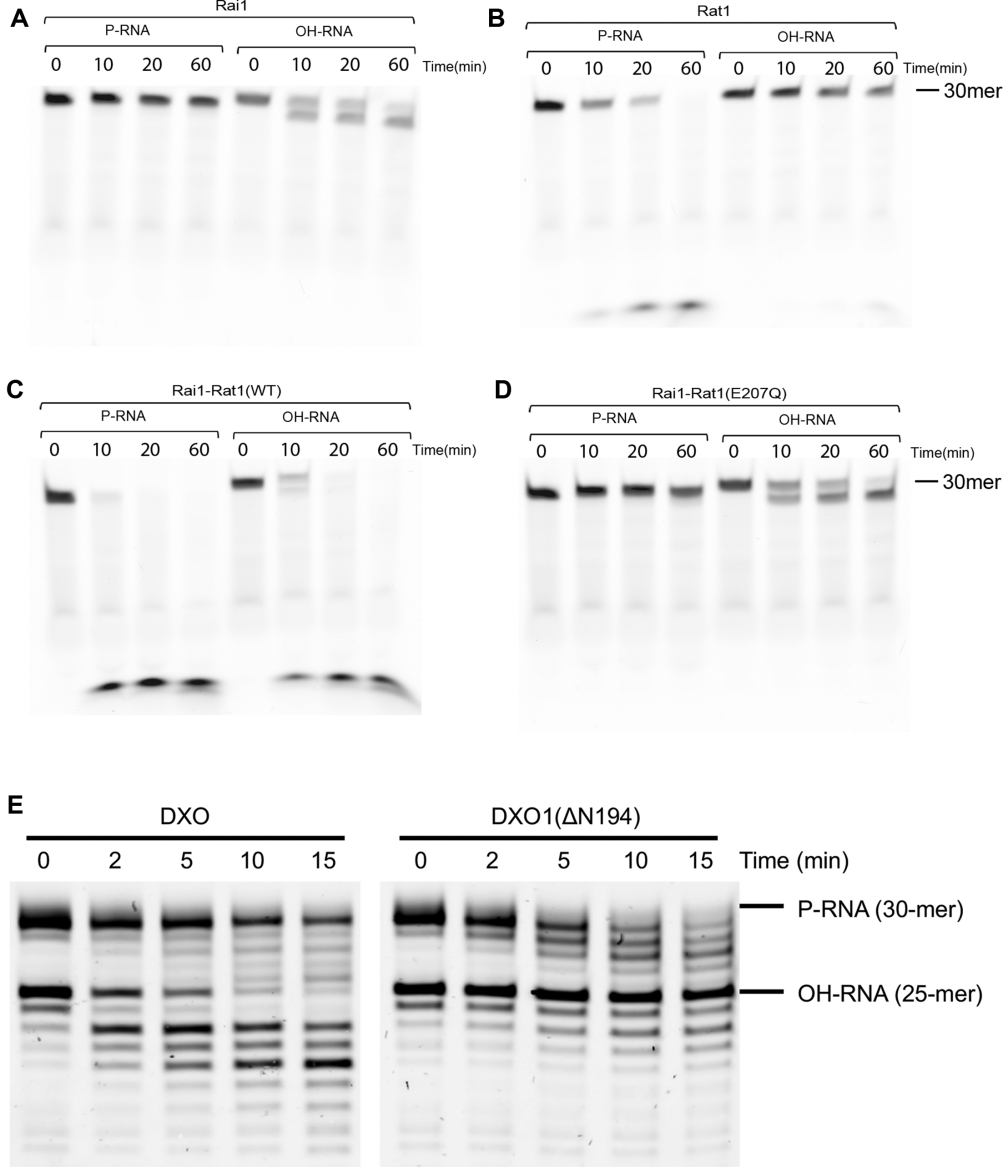
DXO homologs also have HDH activity. (**A**) Time course of the 5′-3′ exoribonuclease activity of 5 nM wild-type (WT) *S. pombe* Rai1 toward 5′-PO_4_ or 5′-OH RNA. (**B**) Same as A but with WT *S. pombe* Rat1 alone. (**C**) Same as in A but with wild-type Rat1-Rai1 complex. (**D**) Same as in A but with a complex of WT Rai1 and catalytic inactive Rat1 (E207Q). (**E**) Simultaneous incubation of 400 nM mouse DXO or *Arabidopsis* DXO1(ΔN194) with 30-mer 5′-PO_4_ RNA and a 25-mer 5′-OH RNA.

Similar to DXO, SpRai1 has PPH, decapping and deNADding activities, but lacks 5′-PO_4_ 5′-3′ exoribonuclease activity (12,13,20). To assess whether SpRai1 also has HDH activity, we compared the decay of 5′-PO_4_ and 5′-OH RNAs by Rai1 and the Rat1–Rai1 complex *in vitro*. Unlike DXO, SpRai1 did not display any activity toward 5′-PO_4_ RNA under the condition tested but could perform a single cleavage reaction on 5′-OH RNA with no further decay of the resultant RNA ( Figure 5A), confirming that it has HDH activity. On the other hand, Rat1 alone displayed processive activity toward 5′-PO_4_ but showed no activity toward 5′-OH RNA (Figure 5B). Consistent with previous studies, this activity was enhanced in the presence of Rai1 (Figure 5C). Interestingly, the Rat1-Rai1 complex also showed robust activity toward 5′-OH RNA (Figure 5C), while Rat1–Rai1 with the catalytically inactive Rat1 mutant E205Q displayed the same activity as Rai1 alone (Figure 5D). These data demonstrate that in the context of the Rat1–Rai1 complex, Rai1 only processes the terminal nucleotide(s) of 5′-OH RNA, while Rat1 is responsible for decay of 5′-PO_4_ RNA.

We also found that the *Arabidopsis* homolog AtDXO1 is active toward the 5′-OH RNA substrate. We showed earlier that the DXO1(ΔN194) variant, which lacks the N-terminal 194 plant-specific amino acids, has PPH activity (19). We tested this variant against a mixture of two RNA substrates containing 5′-PO_4_ or 5′-OH. DXO1(ΔN194) was also able to hydrolyze 5′-OH RNA, but, in contrast to mouse DXO, it had a reduced activity toward this substrate and showed a preference for 5′-PO_4_ RNA (Figure 5E).

## DISCUSSION

**Figure 6.**
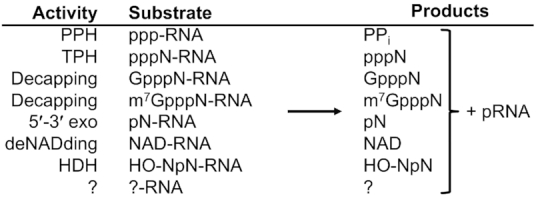
The diverse catalytic activity of DXO/Rai1 enzymes. The various catalytic activities of the DXO/Rai1 family enzymes are shown. They use the same catalytic machinery and share a common product, 5′-PO_4_ RNA (pRNA). Additional activities toward other 5′-end modified RNAs are possible as well, indicated by the question marks.

DXO has previously been shown to possess PPH, decapping, and deNADding activities, and it is unique among the decapping enzymes as it can also completely degrade RNA using its 5′-PO_4_-dependent, distributive 5′-3′ exoribonuclease activity (12,15,20). Here we report an additional activity for the DXO/Rai1 family of enzymes, which is catalyzing the hydrolysis of 5′-OH RNA. DXO initially removes a dinucleotide from 5′-OH RNA, and we propose the name 5′-hydroxyl dinucleotide hydrolase (HDH) for this novel activity. These observations buttress the idea of DXO as a multi-purpose eraser of RNA 5′-end modifications. On the other hand, these distinct activities all share the same catalytic machinery and generate 5′-PO_4_ RNA (pRNA) as a common product (Figure 6), which can be further degraded by DXO and Xrn1/Xrn2. At the same time, DXO does display some 5′-end specificity (at least with respect to decapping and exoribonuclease activities), as RNAs with a 2′-*O*-methyl cap (cap1 and cap2) cannot be degraded (30).

5′-OH RNA is known to exist in eukaryotes, resulting from the activity of some endoribonucleases, self-cleaving ribozymes and intramolecular phosphodiester cleavage (31–36). 5′-OH RNA are intermediates in critical pathways such as ribosomal and transfer RNA processing, and in quality control pathways such as No-Go decay (NGD) (37–39). It has been thought that the decay of some of these 5′-OH RNAs involves phosphorylation by RNA kinases to produce 5′-PO_4_ RNAs, although it is not known if this mechanism applies to all such RNAs. Our studies suggest that DXO and Rat1-Rai1 may also be involved in the removal of such 5′-OH RNAs in cells. In fact an active-site mutant of the cytoplasmic Dxo1 in yeast stabilizes a 5′-OH RNA intermediate in the NGD pathway (39), and Rai1 has some Rat1-independent RNA processing activities which could be related to its HDH activity (40). Notably, 5'-OH cleavage products from pre-rRNA may also be further processed by Rai1 HDH activity, since endonuclease Las1 responsible for this cleavage has been reported to act in a complex with Rai1 (39). Previously, only Rrp17 (Nol12 in humans), which is involved in rRNA processing, was shown to have 5′-OH RNA exoribonuclease activity in eukaryotes (41). Together, our studies have uncovered an additional route for 5′-3′ eukaryotic RNA decay.

An interesting question arises as to the fate of the 5′-OH dinucleotide that is released by DXO from 5′-OH RNA. Dinucleotide nucleases, for example Orn in bacteria (42) and Rexo2 in humans (43), can degrade such oligos in a 3′-5′ direction. Therefore, it would be interesting to find out if Rexo2 and related enzymes could mediate the degradation of the 5′-OH dinucleotide product from DXO.

Our crystal structure of DXO with 5′-OH RNA provides the molecular basis for the HDH activity, and it may also explain why this activity can be higher than that toward 5′-PO_4_ RNA. By accommodating an additional nucleotide 5′ to the scissile phosphate compared to 5′-PO_4_ RNA, DXO can make further contacts to 5′-OH RNA, mainly through a stacking interaction of the terminal base with the highly conserved Trp131 residue. The *syn* configuration of the next base may also increase binding of DXO to 5′-OH RNA since the conserved Tyr189 can then pack between U_1_ and U_2_ in this conformation. Finally, the 5′-OH group is positioned against the U_1_ base thus discriminating against a terminal 5′-PO4.

DXO activity toward 5′-OH RNA was previously tested but the enzyme was found to be inactive (15). This discrepancy may be due to differences in the RNA sequences used for our structural and biochemical analyses *versus* previous studies (uracil and adenosine versus guanosine). Our assays here indicate that activity toward 5′-OH RNA appears to be strongly sequence-dependent and in substrates lacking G at their 5′ ends the 5′-OH RNA is preferentially digested in comparison to the 5′-PO_4_ RNA. The decreased activity toward RNA with 5′ Gs may explain why DXO was previously reported to be inactive toward 5′-OH RNA (15). On the other hand, U and C are hydrolyzed faster than A suggesting that DXO is more active toward pyrimidines than purines at the 5′ end. The exact mechanism for this sequence preference by DXO will require further structural and biochemical studies to illuminate. It might be possible that a 5′-OH GG dinucleotide is docked into the active site in a less optimal conformation than a 5′-PO_4_ GG dinucleotide.

We demonstrate that SpRai1 and AtDXO1 can also degrade 5′-OH RNA, thereby extending the HDH activity to other DXO homologs. Similar to DXO, SpRai1 has a strong preference for 5′-OH RNA, although AtDXO1 prefers 5′-PO_4_ RNA. The differing activities of DXO homologs toward these RNAs further demonstrate the diversity of activities within the DXO/Rai1 enzyme family and suggest that the extent of degradation of 5′-OH RNAs *in vivo* may vary depending on the organism. At least in *S. pombe*, the HDH activity of SpRai1 licenses the Rat1–Rai1 complex the ability to completely degrade 5′-OH RNA, which is a novel activity for Rat1–Rai1 and could lead to uncovering new functions for this complex.

## Supplementary Material

gkz1107_Supplemental_File

## References

[1] Ghosh A., Lima C.D. Enzymology of RNA cap synthesis. Wiley Interdiscip. Rev. RNA. 2010; 1:152–172. 21956912 10.1002/wrna.19 PMC3962952

[2] Mauer J., Luo X., Blanjoie A., Jiao X., Grozhik A.V., Patil D.P., Linder B., Pickering B.F., Vasseur J.J., Chen Q. et al. Reversible methylation of m(6)Am in the 5′ cap controls mRNA stability. Nature. 2017; 541:371–375. 28002401 10.1038/nature21022 PMC5513158

[3] Kiledjian M. Eukaryotic RNA 5′-end NAD(+) capping and deNADding. Trends Cell Biol. 2018; 28:454–464. 29544676 10.1016/j.tcb.2018.02.005 PMC5962413

[4] Kramer S., McLennan A.G. The complex enzymology of mRNA decapping: enzymes of four classes cleave pyrophosphate bonds. Wiley Interdiscip. Rev. RNA. 2019; 10:e1511. 30345629 10.1002/wrna.1511

[5] Julius C., Yuzenkova Y. Noncanonical RNA-capping: discovery, mechanism and physiological role debate. Wiley Interdiscip. Rev. RNA. 2019; 10:e1512. 30353673 10.1002/wrna.1512

[6] Meyer S., Temme C., Wahle E. Messenger RNA turnover in eukaryotes: pathways and enzymes. Crit. Rev. Biochem. Mol. Biol. 2004; 39:197–216. 15596551 10.1080/10409230490513991

[7] Garneau N.L., Wilusz J., Wilusz C.J. The highways and byways of mRNA decay. Nat. Rev. Mol. Cell Biol. 2007; 8:113–126. 17245413 10.1038/nrm2104

[8] Houseley J., Tollervey D. The many pathways of RNA degradation. Cell. 2009; 136:763–776. 19239894 10.1016/j.cell.2009.01.019

[9] Song M.G., Bail S., Kiledjian M. Mutiple Nudix family proteins possess mRNA decapping activity. RNA. 2013; 19:390–399. 23353937 10.1261/rna.037309.112 PMC3677249

[10] Parker R., Song H. The enzymes and control of eukaryotic mRNA turnover. Nat. Struct. Mol. Biol. 2004; 11:121–127. 14749774 10.1038/nsmb724

[11] Chang J.H., Xiang S., Tong L. Structures of 5′-3′ exoribonucleases. Enzymes. 2012; 31:115–129. 27166443 10.1016/B978-0-12-404740-2.00006-9

[12] Xiang S., Cooper-Morgan A., Jiao X., Kiledjian M., Manley J.L., Tong L. Structure and function of the 5′→3′ exoribonuclease Rat1 and its activating partner Rai1. Nature. 2009; 458:784–788. 19194460 10.1038/nature07731 PMC2739979

[13] Jiao X., Xiang S., Oh C.-S., Martin C.E., Tong L., Kiledjian M. Identification of a quality-control mechanism for mRNA 5′-end capping. Nature. 2010; 467:608–611. 20802481 10.1038/nature09338 PMC2948066

[14] Chang J.H., Jiao X., Chiba K., Kiledjian M., Tong L. Dxo1 is a new type of eukaryotic enzyme with both decapping and 5′-3′ exoribonuclease activity. Nature Struct. Mol. Biol. 2012; 19:1011–1017. 22961381 10.1038/nsmb.2381 PMC3711404

[15] Jiao X., Chang J.H., Kilic T., Tong L., Kiledjian M. A mammalian pre-mRNA 5′ end capping quality control mechanism and an unexpected link of capping to pre-mRNA processing. Mol. Cell. 2013; 50:104–115. 23523372 10.1016/j.molcel.2013.02.017 PMC3630477

[16] Stevens A., Poole T.L. 5′-exonuclease-2 of Saccharomyces cerevisiae. Purification and features of ribonuclease activity with comparison to 5′-exonuclease-1. J. Biol. Chem. 1995; 270:16063–16069. 7608167 10.1074/jbc.270.27.16063

[17] Xue Y., Bai X., Lee I., Kallstrom G., Ho J., Brown J., Stevens A., Johnson A.W. Saccharomyces cerevisiae RAI1 (YGL246c) is homologous to human DOM3Z and encodes a protein that binds the nuclear exoribonuclease Rat1p. Mol. Cell Biol. 2000; 20:4006–4015. 10805743 10.1128/mcb.20.11.4006-4015.2000 PMC85771

[18] Wang V.Y., Jiao X., Kiledjian M., Tong L. Structural and biochemical studies of the distinct activity profiles of Rai1 enzymes. Nucleic Acids Res. 2015; 43:6596–6606. 26101253 10.1093/nar/gkv620 PMC4513879

[19] Kwasnik A., Wang V.Y., Krzyszton M., Gozdek A., Zakrzewska-Placzek M., Stepniak K., Poznanski J., Tong L., Kufel J. Arabidopsis DXO1 links RNA turnover and chloroplast function independently of its enzymatic activity. Nucleic Acids Res. 2019; 47:4751–4764. 30949699 10.1093/nar/gkz100 PMC6511851

[20] Jiao X., Doamekpor S.K., Bird J.G., Nickels B.E., Tong L., Hart R.P., Kiledjian M. 5′ end nicotinamide adenine dinucleotide cap in human cells promotes RNA decay through DXO-mediated deNADding. Cell. 2017; 168:1015–1027. 28283058 10.1016/j.cell.2017.02.019 PMC5371429

[21] Chen Y.G., Kowtoniuk W.E., Agarwal I., Shen Y., Liu D.R. LC/MS analysis of cellular RNA reveals NAD-linked RNA. Nat. Chem. Biol. 2009; 5:879–881. 19820715 10.1038/nchembio.235 PMC2842606

[22] Cahova H., Winz M.L., Hofer K., Nubel G., Jaschke A. NAD captureSeq indicates NAD as a bacterial cap for a subset of regulatory RNAs. Nature. 2015; 519:374–377. 25533955 10.1038/nature14020

[23] Vvedenskaya I.O., Bird J.G., Zhang Y., Zhang Y., Jiao X., Barvik I., Krasny L., Kiledjian M., Taylor D.M., Ebright R.H. et al. CapZyme-Seq comprehensively defines promoter-sequence determinants for RNA 5′ capping with NAD. Mol. Cell. 2018; 70:553–564. 29681497 10.1016/j.molcel.2018.03.014 PMC5935523

[24] Bird J.G., Zhang Y., Tian Y., Panova N., Barvik I., Greene L., Liu M., Buckley B., Krasny L., Lee J.K. et al. The mechanism of RNA 5′ capping with NAD+, NADH and desphospho-CoA. Nature. 2016; 535:444–447. 27383794 10.1038/nature18622 PMC4961592

[25] Walters R.W., Matheny T., Mizoue L.S., Rao B.S., Muhlrad D., Parker R. Identification of NAD+ capped mRNAs in Saccharomyces cerevisiae. Proc. Natl. Acad. Sci. U.S.A. 2017; 114:480–485. 28031484 10.1073/pnas.1619369114 PMC5255579

[26] Kabsch W. Integration, scaling, space-group assignment and post-refinement. Acta Cryst. 2010; D66:133–144. 10.1107/S0907444909047374 PMC2815666 20124693

[27] Adams P.D., Grosse-Kunstleve R.W., Hung L.-W., Ioerger T.R., McCoy A.J., Moriarty N.W., Read R.J., Sacchettini J.C., Sauter N.K., Terwilliger T.C. PHENIX: building a new software for automated crystallographic structure determination. Acta Cryst. 2002; D58:1948–1954. 10.1107/s0907444902016657 12393927

[28] Emsley P., Cowtan K.D. Coot: model-building tools for molecular graphics. Acta Cryst. 2004; D60:2126–2132. 10.1107/S0907444904019158 15572765

[29] Sinturel F., Pellegrini O., Xiang S., Tong L., Condon C., Benard L. Real-time fluorescence detection of exoribonucleases. RNA. 2009; 15:2057–2062. 19767421 10.1261/rna.1670909 PMC2764478

[30] Picard-Jean F., Brand C., Tremblay-Letourneau M., Allaire A., Beaudoin M.C., Boudreault S., Duval C., Rainville-Sirois J., Robert F., Pelletier J. et al. 2′-O-methylation of the mRNA cap protects RNAs from decapping and degradation by DXO. PLoS One. 2018; 13:e0193804. 29601584 10.1371/journal.pone.0193804 PMC5877831

[31] Trotta C.R., Miao F., Arn E.A., Stevens S.W., Ho C.K., Rauhut R., Abelson J.N. The yeast tRNA splicing endonuclease: a tetrameric enzyme with two active site subunits homologous to the archaeal tRNA endonucleases. Cell. 1997; 89:849–858. 9200603 10.1016/s0092-8674(00)80270-6

[32] Soukup G.A., Breaker R.R. Relationship between internucleotide linkage geometry and the stability of RNA. RNA. 1999; 5:1308–1325. 10573122 10.1017/s1355838299990891 PMC1369853

[33] Ferre-D’Amare A.R., Scott W.G. Small self-cleaving ribozymes. Cold Spring Harbor Perspect. Biol. 2010; 2:a003574. 10.1101/cshperspect.a003574 PMC2944367 20843979

[34] Luhtala N., Parker R. T2 family ribonucleases: ancient enzymes with diverse roles. Trends Biochem. Sci. 2010; 35:253–259. 20189811 10.1016/j.tibs.2010.02.002 PMC2888479

[35] Roth A., Weinberg Z., Chen A.G., Kim P.B., Ames T.D., Breaker R.R. A widespread self-cleaving ribozyme class is revealed by bioinformatics. Nat. Chem. Biol. 2014; 10:56–60. 24240507 10.1038/nchembio.1386 PMC3867598

[36] Peach S.E., York K., Hesselberth J.R. Global analysis of RNA cleavage by 5′-hydroxyl RNA sequencing. Nucleic Acids Res. 2015; 43:e108. 26001965 10.1093/nar/gkv536 PMC4787814

[37] Lopes R.R., Kessler A.C., Polycarpo C., Alfonzo J.D. Cutting, dicing, healing and sealing: the molecular surgery of tRNA. Wiley Interdiscip. Rev. RNA. 2015; 6:337–349. 25755220 10.1002/wrna.1279 PMC4397177

[38] Tomecki R., Sikorski P.J., Zakrzewska-Placzek M. Comparison of preribosomal RNA processing pathways in yeast, plant and human cells - focus on coordinated action of endo- and exoribonucleases. FEBS Lett. 2017; 591:1801–1850. 28524231 10.1002/1873-3468.12682

[39] Navickas A., Chamois S., Saint-Fort R., Henri J., Torchet C., Benard L. A unique No-Go decay cleavage in mRNA exit-tunnel of ribosome produces 5′-OH ends phosphorylated by RlgI. 2018; bioRxiv doi:08 November 2018, preprint: not peer reviewed 10.1101/465633. PMC6949252 31913314

[40] Schillewaert S., Wacheul L., Lhomme F., Lafontaine D.L. The evolutionarily conserved protein Las1 is required for pre-rRNA processing at both ends of ITS2. Mol. Cell Biol. 2012; 32:430–444. 22083961 10.1128/MCB.06019-11 PMC3255765

[41] Oeffinger M., Zenklusen D., Ferguson A., Wei K.E., El Hage A., Chait B.T., Singer R.H., Rout M.P. Rrp17p is a eukaryotic exonuclease required for 5′ end processing of pre-60S ribosomal RNA. Mol. Cell. 2009; 36:768–781. 20005841 10.1016/j.molcel.2009.11.011 PMC2806520

[42] Kim S.K., Lormand J.D., Weiss C.A., Eger K.A., Turdiev H., Turdiev A., Winkler W.C., Sondermann H., Lee V.T. A dedicated diribonucleotidase resolves a key bottleneck for the terminal step of RNA degradation. eLife. 2019; 8:e46313. 31225796 10.7554/eLife.46313 PMC6613908

[43] Chu L.Y., Agrawal S., Chen Y.P., Yang W.Z., Yuan H.S. Structural insights into nanoRNA degradation by human Rexo2. RNA. 2019; 25:737–746. 30926754 10.1261/rna.070557.119 PMC6521605

