# Correction to ‘A novel 5′-hydroxyl dinucleotide hydrolase activity for the DXO/Rai1 family of enzymes’

**DOI:** 10.1093/nar/gkab570

**Published:** 2021-06-24

**Authors:** Selom K Doamekpor, Agnieszka Gozdek, Aleksandra Kwasnik, Joanna Kufel, Liang Tong

**Affiliations:** Department of Biological Sciences, Columbia University, New York, NY 10027, USA; Institute of Genetics and Biotechnology, Faculty of Biology, University of Warsaw, 02-106 Warsaw, Poland; Institute of Genetics and Biotechnology, Faculty of Biology, University of Warsaw, 02-106 Warsaw, Poland; Institute of Genetics and Biotechnology, Faculty of Biology, University of Warsaw, 02-106 Warsaw, Poland; Department of Biological Sciences, Columbia University, New York, NY 10027, USA

The authors wish to add a source of funding, shown in bold, to their article (1).
