# Supplementary material for: A novel 5′-hydroxyl dinucleotide hydrolase activity for the DXO/Rai1 family of enzymes
Source: Nucleic Acids Res. 2019 Nov 28;48(1):349–58. doi: 10.1093/nar/gkz1107 (PMC6943137; doi:10.1093/nar/gkz1107)
Supplement: gkz1107_Supplemental_File [file gkz1107_supplemental_file.pdf]

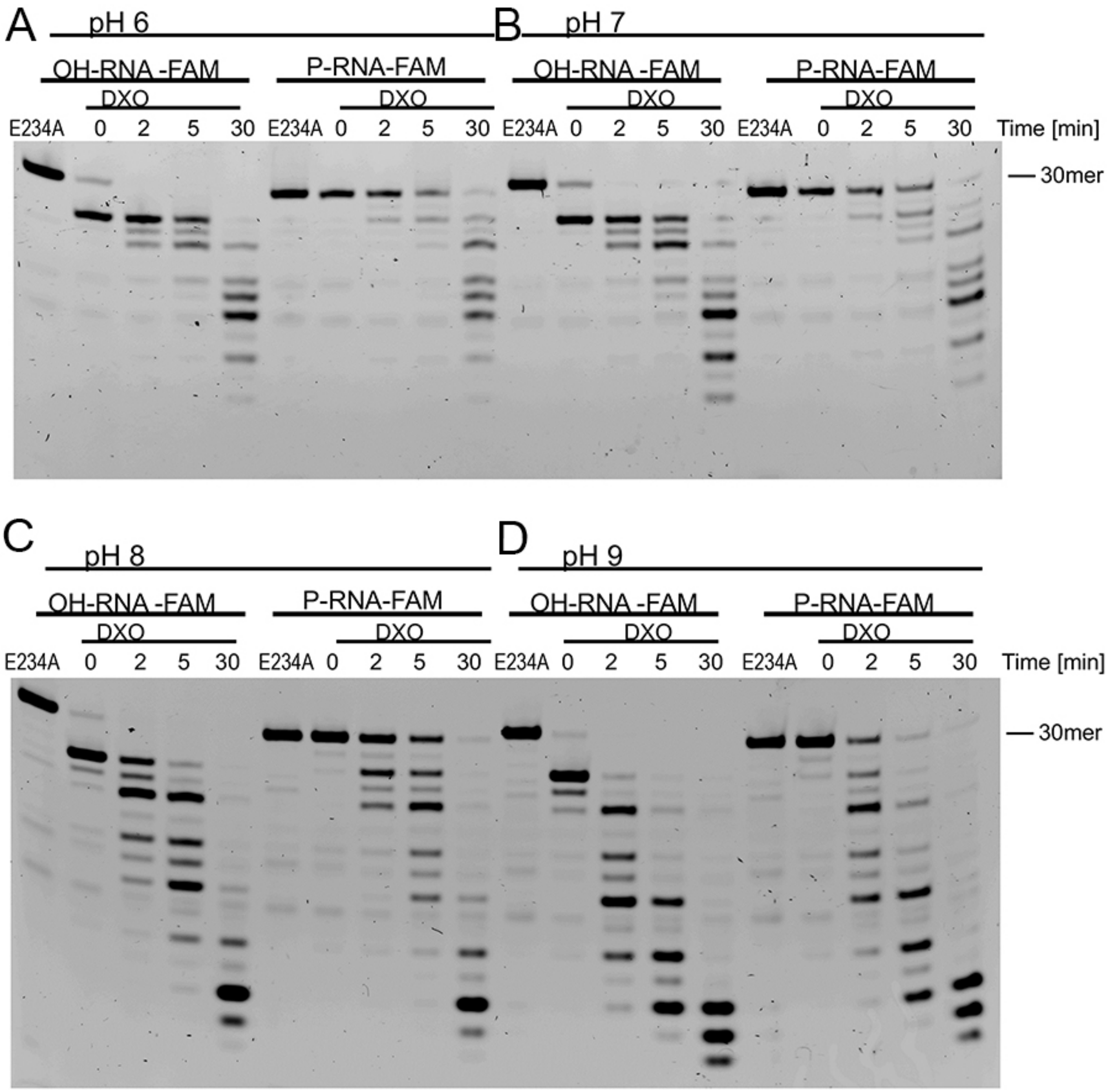

Fig. S1. HDH and exoribonuclease activity of DXO at different pH. Time course of DXO activity toward 5′-OH and 5′-PO_4 FAM-labeled G-less substrates at pH 6 (A), 7 (B), 8 (C) and 9 (D). Enzyme and substrate concentrations are 100 nM.
